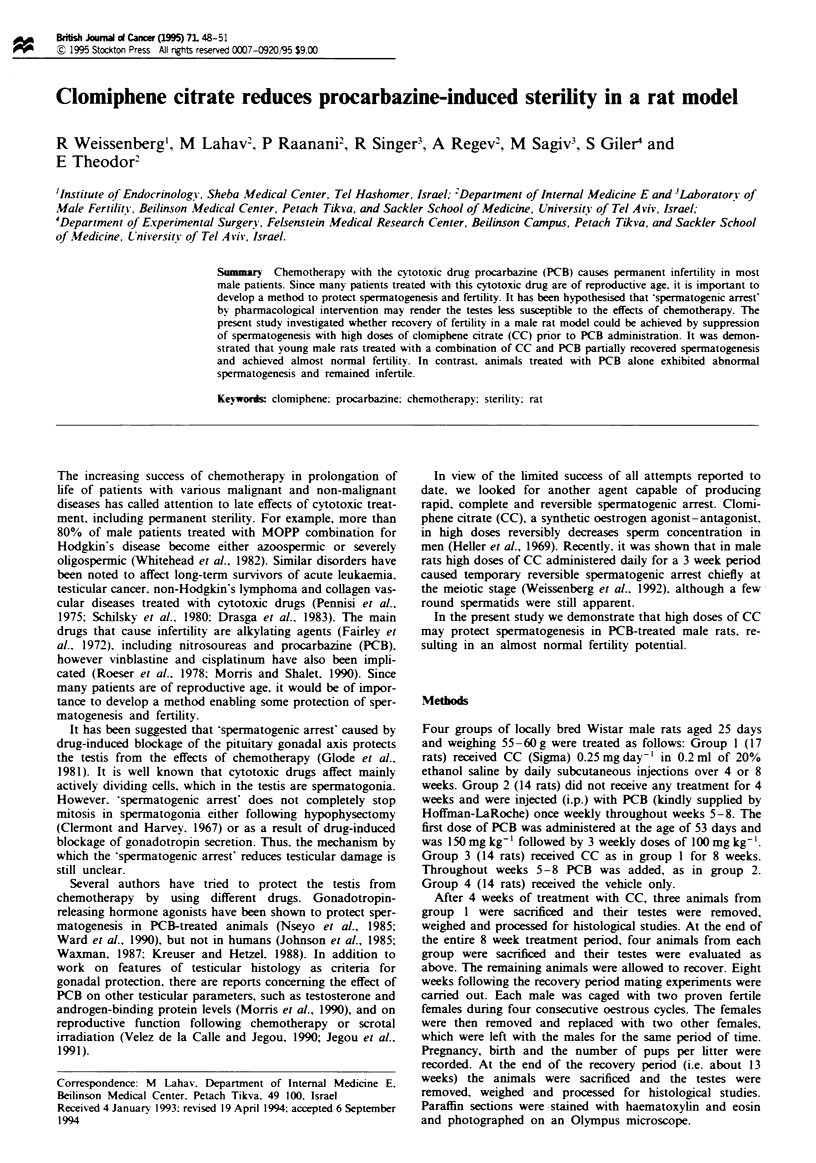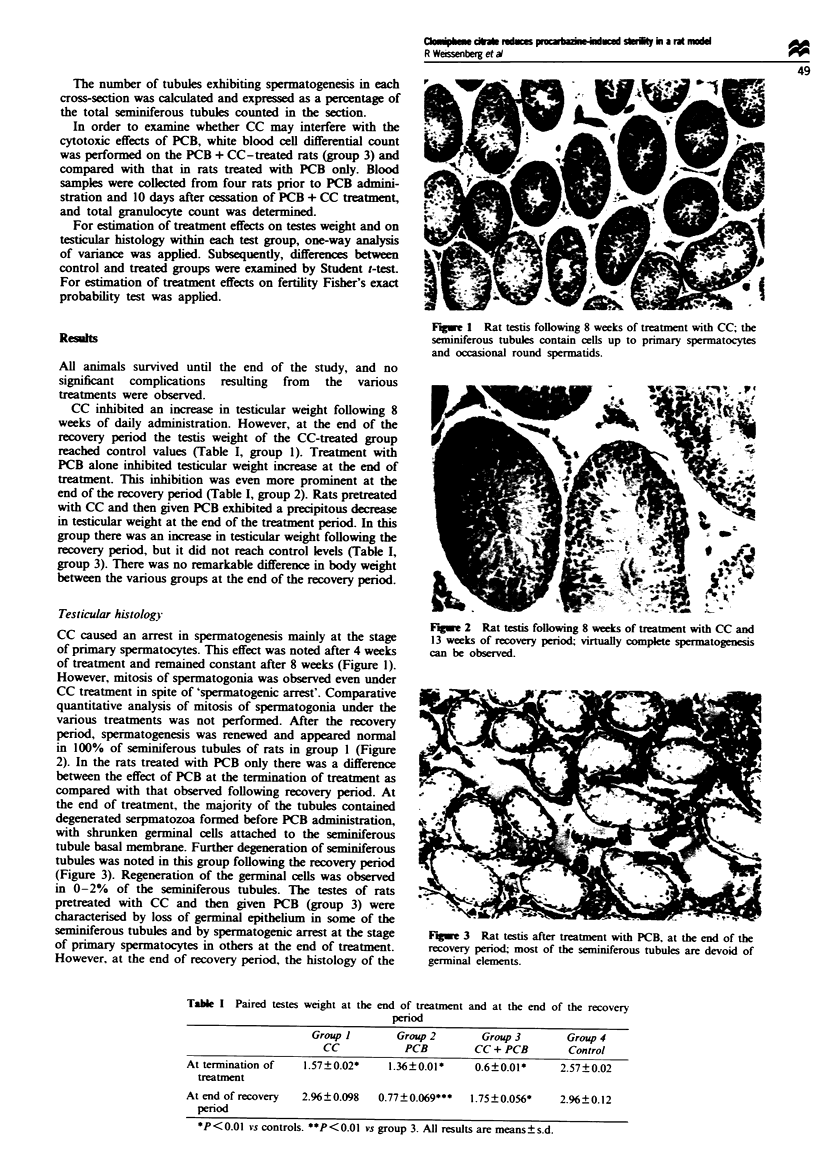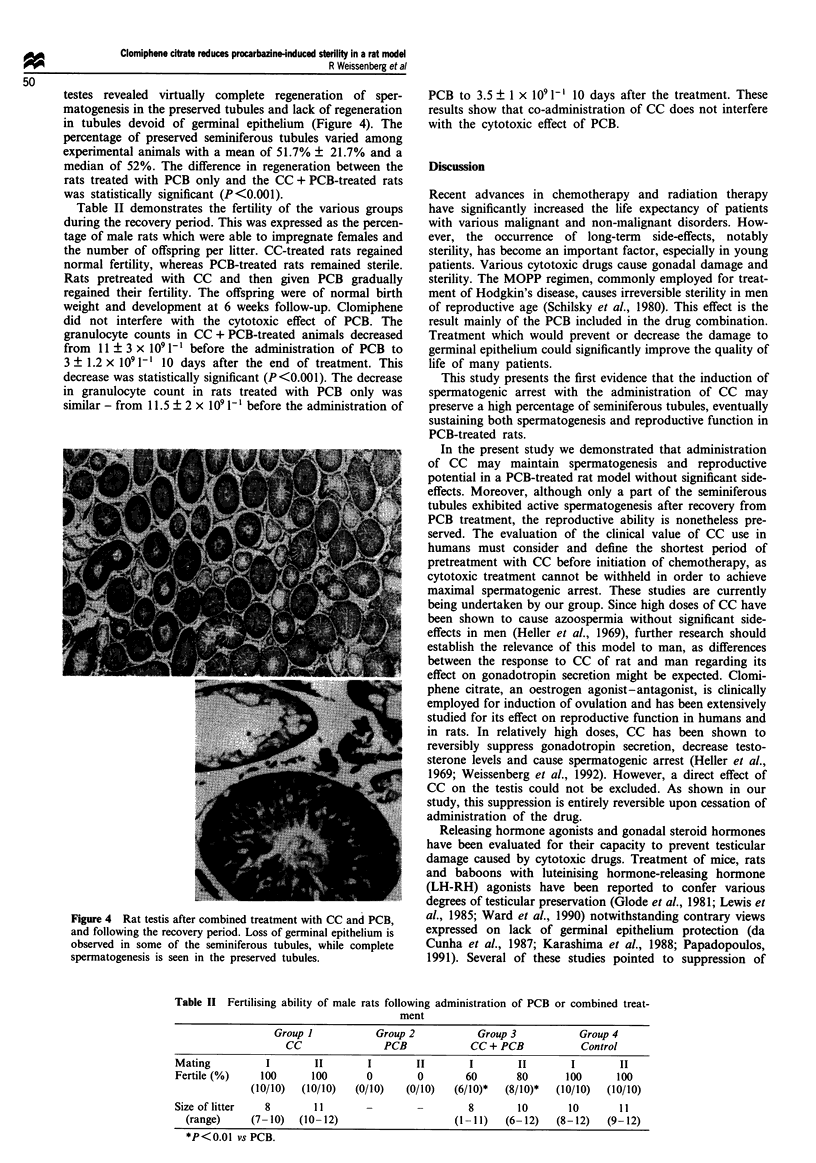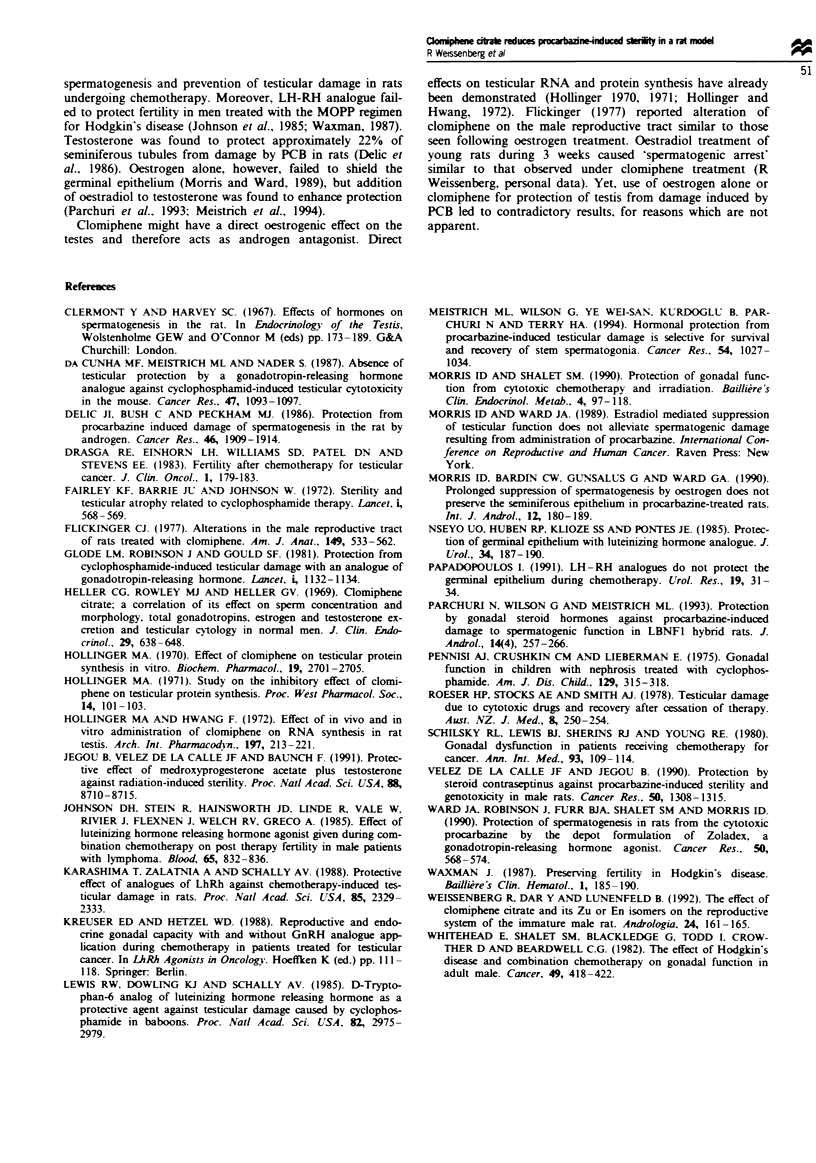# Clomiphene citrate reduces procarbazine-induced sterility in a rat model.

**DOI:** 10.1038/bjc.1995.10

**Published:** 1995-01

**Authors:** R. Weissenberg, M. Lahav, P. Raanani, R. Singer, A. Regev, M. Sagiv, S. Giler, E. Theodor

**Affiliations:** Institute of Endocrinology, Sheba Medical Center, Tel Hashomer, Israel.

## Abstract

**Images:**


British Journal of Cancer (1995) 71, 48-51

© 1995 Stockton Press All rights reserved 0007-0920/95 $9.00

### Clomiphene citrate reduces procarbazine-induced sterility in a rat model

R Weissenberg[1], M Lahav[2], P Raanani[2], R Singer[3], A Regev[2], M Sagiv[3], S Giler[4] and E Theodor[2]

[1]*Institute of Endocrinology, Sheba Medical Center, Tel Hashomer, Israel;* [2]*Department of Internal Medicine E and* [3]*Laboratory of Male Fertility, Beilinson Medical Center, Petach Tikva, and Sackler School of Medicine, University of Tel Aviv, Israel;* [4]*Department of Experimental Surgery, Felsenstein Medical Research Center, Beilinson Campus, Petach Tikva, and Sackler School of Medicine, University of Tel Aviv, Israel.*

**Summary** Chemotherapy with the cytotoxic drug procarbazine (PCB) causes permanent infertility in most male patients. Since many patients treated with this cytotoxic drug are of reproductive age, it is important to develop a method to protect spermatogenesis and fertility. It has been hypothesised that 'spermatogenic arrest' by pharmacological intervention may render the testes less susceptible to the effects of chemotherapy. The present study investigated whether recovery of fertility in a male rat model could be achieved by suppression of spermatogenesis with high doses of clomiphene citrate (CC) prior to PCB administration. It was demonstrated that young male rats treated with a combination of CC and PCB partially recovered spermatogenesis and achieved almost normal fertility. In contrast, animals treated with PCB alone exhibited abnormal spermatogenesis and remained infertile.

**Keywords:** clomiphene; procarbazine; chemotherapy; sterility; rat

The increasing success of chemotherapy in prolongation of life of patients with various malignant and non-malignant diseases has called attention to late effects of cytotoxic treatment, including permanent sterility. For example, more than 80% of male patients treated with MOPP combination for Hodgkin's disease become either azoospermic or severely oligospermic (Whitehead *et al.*, 1982). Similar disorders have been noted to affect long-term survivors of acute leukaemia, testicular cancer, non-Hodgkin's lymphoma and collagen vascular diseases treated with cytotoxic drugs (Pennisi *et al.*, 1975; Schilsky *et al.*, 1980; Drasga *et al.*, 1983). The main drugs that cause infertility are alkylating agents (Fairley *et al.*, 1972), including nitrosoureas and procarbazine (PCB), however vinblastine and cisplatinum have also been implicated (Roeser *et al.*, 1978; Morris and Shalet, 1990). Since many patients are of reproductive age, it would be of importance to develop a method enabling some protection of spermatogenesis and fertility.

It has been suggested that 'spermatogenic arrest' caused by drug-induced blockage of the pituitary gonadal axis protects the testis from the effects of chemotherapy (Glode *et al.*, 1981). It is well known that cytotoxic drugs affect mainly actively dividing cells, which in the testis are spermatogonia. However, 'spermatogenic arrest' does not completely stop mitosis in spermatogonia either following hypophysectomy (Clermont and Harvey, 1967) or as a result of drug-induced blockage of gonadotropin secretion. Thus, the mechanism by which the 'spermatogenic arrest' reduces testicular damage is still unclear.

Several authors have tried to protect the testis from chemotherapy by using different drugs. Gonadotropin-releasing hormone agonists have been shown to protect spermatogenesis in PCB-treated animals (Nseyo *et al.*, 1985; Ward *et al.*, 1990), but not in humans (Johnson *et al.*, 1985; Waxman, 1987; Kreuser and Hetzel, 1988). In addition to work on features of testicular histology as criteria for gonadal protection, there are reports concerning the effect of PCB on other testicular parameters, such as testosterone and androgen-binding protein levels (Morris *et al.*, 1990), and on reproductive function following chemotherapy or scrotal irradiation (Velez de la Calle and Jegou, 1990; Jegou *et al.*, 1991).

Correspondence: M Lahav, Department of Internal Medicine E, Beilinson Medical Center, Petach Tikva, 49 100, Israel
Received 4 January 1993; revised 19 April 1994; accepted 6 September 1994

In view of the limited success of all attempts reported to date, we looked for another agent capable of producing rapid, complete and reversible spermatogenic arrest. Clomiphene citrate (CC), a synthetic oestrogen agonist-antagonist, in high doses reversibly decreases sperm concentration in men (Heller *et al.*, 1969). Recently, it was shown that in male rats high doses of CC administered daily for a 3 week period caused temporary reversible spermatogenic arrest chiefly at the meiotic stage (Weissenberg *et al.*, 1992), although a few round spermatids were still apparent.

In the present study we demonstrate that high doses of CC may protect spermatogenesis in PCB-treated male rats, resulting in an almost normal fertility potential.

### Methods

Four groups of locally bred Wistar male rats aged 25 days and weighing 55-60 g were treated as follows: Group 1 (17 rats) received CC (Sigma) 0.25 mg $day^{-1}$ in 0.2 ml of 20% ethanol saline by daily subcutaneous injections over 4 or 8 weeks. Group 2 (14 rats) did not receive any treatment for 4 weeks and were injected (i.p.) with PCB (kindly supplied by Hoffman-LaRoche) once weekly throughout weeks 5-8. The first dose of PCB was administered at the age of 53 days and was 150 mg $kg^{-1}$ followed by 3 weekly doses of 100 mg $kg^{-1}$. Group 3 (14 rats) received CC as in group 1 for 8 weeks. Throughout weeks 5-8 PCB was added, as in group 2. Group 4 (14 rats) received the vehicle only.

After 4 weeks of treatment with CC, three animals from group 1 were sacrificed and their testes were removed, weighed and processed for histological studies. At the end of the entire 8 week treatment period, four animals from each group were sacrificed and their testes were evaluated as above. The remaining animals were allowed to recover. Eight weeks following the recovery period mating experiments were carried out. Each male was caged with two proven fertile females during four consecutive oestrous cycles. The females were then removed and replaced with two other females, which were left with the males for the same period of time. Pregnancy, birth and the number of pups per litter were recorded. At the end of the recovery period (i.e. about 13 weeks) the animals were sacrificed and the testes were removed, weighed and processed for histological studies. Paraffin sections were stained with haematoxylin and eosin and photographed on an Olympus microscope.

The number of tubules exhibiting spermatogenesis in each cross-section was calculated and expressed as a percentage of the total seminiferous tubules counted in the section.

In order to examine whether CC may interfere with the cytotoxic effects of PCB, white blood cell differential count was performed on the PCB + CC-treated rats (group 3) and compared with that in rats treated with PCB only. Blood samples were collected from four rats prior to PCB administration and 10 days after cessation of PCB + CC treatment, and total granulocyte count was determined.

For estimation of treatment effects on testes weight and on testicular histology within each test group, one-way analysis of variance was applied. Subsequently, differences between control and treated groups were examined by Student $t$-test. For estimation of treatment effects on fertility Fisher's exact probability test was applied.

### Results

All animals survived until the end of the study, and no significant complications resulting from the various treatments were observed.

CC inhibited an increase in testicular weight following 8 weeks of daily administration. However, at the end of the recovery period the testis weight of the CC-treated group reached control values (Table I, group 1). Treatment with PCB alone inhibited testicular weight increase at the end of treatment. This inhibition was even more prominent at the end of the recovery period (Table I, group 2). Rats pretreated with CC and then given PCB exhibited a precipitous decrease in testicular weight at the end of the treatment period. In this group there was an increase in testicular weight following the recovery period, but it did not reach control levels (Table I, group 3). There was no remarkable difference in body weight between the various groups at the end of the recovery period.

### *Testicular histology*

CC caused an arrest in spermatogenesis mainly at the stage of primary spermatocytes. This effect was noted after 4 weeks of treatment and remained constant after 8 weeks (Figure 1). However, mitosis of spermatogonia was observed even under CC treatment in spite of 'spermatogenic arrest'. Comparative quantitative analysis of mitosis of spermatogonia under the various treatments was not performed. After the recovery period, spermatogenesis was renewed and appeared normal in 100% of seminiferous tubules of rats in group 1 (Figure 2). In the rats treated with PCB only there was a difference between the effect of PCB at the termination of treatment as compared with that observed following recovery period. At the end of treatment, the majority of the tubules contained degenerated serpmatozoa formed before PCB administration, with shrunken germinal cells attached to the seminiferous tubule basal membrane. Further degeneration of seminiferous tubules was noted in this group following the recovery period (Figure 3). Regeneration of the germinal cells was observed in 0–2% of the seminiferous tubules. The testes of rats pretreated with CC and then given PCB (group 3) were characterised by loss of germinal epithelium in some of the seminiferous tubules and by spermatogenic arrest at the stage of primary spermatocytes in others at the end of treatment. However, at the end of recovery period, the histology of the

**Figure 1** Rat testis following 8 weeks of treatment with CC; the seminiferous tubules contain cells up to primary spermatocytes and occasional round spermatids.

**Figure 2** Rat testis following 8 weeks of treatment with CC and 13 weeks of recovery period; virtually complete spermatogenesis can be observed.

**Figure 3** Rat testis after treatment with PCB, at the end of the recovery period; most of the seminiferous tubules are devoid of germinal elements.

**Table I** Paired testes weight at the end of treatment and at the end of the recovery period

| | *Group 1* *CC* | *Group 2* *PCB* | *Group 3* *CC + PCB* | *Group 4* *Control* |
|---|---|---|---|---|
| At termination of treatment | 1.57±0.02* | 1.36±0.01* | 0.6±0.01* | 2.57±0.02 |
| At end of recovery period | 2.96±0.098 | 0.77±0.069*** | 1.75±0.056* | 2.96±0.12 |

*$P<0.01$ *vs* controls. **$P<0.01$ *vs* group 3. All results are means±s.d.

testes revealed virtually complete regeneration of spermatogenesis in the preserved tubules and lack of regeneration in tubules devoid of germinal epithelium (Figure 4). The percentage of preserved seminiferous tubules varied among experimental animals with a mean of 51.7% ± 21.7% and a median of 52%. The difference in regeneration between the rats treated with PCB only and the CC + PCB-treated rats was statistically significant ($P<0.001$).

Table II demonstrates the fertility of the various groups during the recovery period. This was expressed as the percentage of male rats which were able to impregnate females and the number of offspring per litter. CC-treated rats regained normal fertility, whereas PCB-treated rats remained sterile. Rats pretreated with CC and then given PCB gradually regained their fertility. The offspring were of normal birth weight and development at 6 weeks follow-up. Clomiphene did not interfere with the cytotoxic effect of PCB. The granulocyte counts in CC + PCB-treated animals decreased from $11 \pm 3 \times 10^9 l^{-1}$ before the administration of PCB to $3 \pm 1.2 \times 10^9 l^{-1}$ 10 days after the end of treatment. This decrease was statistically significant ($P<0.001$). The decrease in granulocyte count in rats treated with PCB only was similar – from $11.5 \pm 2 \times 10^9 l^{-1}$ before the administration of PCB to $3.5 \pm 1 \times 10^9 l^{-1}$ 10 days after the treatment. These results show that co-administration of CC does not interfere with the cytotoxic effect of PCB.

**Figure 4** Rat testis after combined treatment with CC and PCB, and following the recovery period. Loss of germinal epithelium is observed in some of the seminiferous tubules, while complete spermatogenesis is seen in the preserved tubules.

### Discussion

Recent advances in chemotherapy and radiation therapy have significantly increased the life expectancy of patients with various malignant and non-malignant disorders. However, the occurrence of long-term side-effects, notably sterility, has become an important factor, especially in young patients. Various cytotoxic drugs cause gonadal damage and sterility. The MOPP regimen, commonly employed for treatment of Hodgkin's disease, causes irreversible sterility in men of reproductive age (Schilsky *et al.*, 1980). This effect is the result mainly of the PCB included in the drug combination. Treatment which would prevent or decrease the damage to germinal epithelium could significantly improve the quality of life of many patients.

This study presents the first evidence that the induction of spermatogenic arrest with the administration of CC may preserve a high percentage of seminiferous tubules, eventually sustaining both spermatogenesis and reproductive function in PCB-treated rats.

In the present study we demonstrated that administration of CC may maintain spermatogenesis and reproductive potential in a PCB-treated rat model without significant side-effects. Moreover, although only a part of the seminiferous tubules exhibited active spermatogenesis after recovery from PCB treatment, the reproductive ability is nonetheless preserved. The evaluation of the clinical value of CC use in humans must consider and define the shortest period of pretreatment with CC before initiation of chemotherapy, as cytotoxic treatment cannot be withheld in order to achieve maximal spermatogenic arrest. These studies are currently being undertaken by our group. Since high doses of CC have been shown to cause azoospermia without significant side-effects in men (Heller *et al.*, 1969), further research should establish the relevance of this model to man, as differences between the response to CC of rat and man regarding its effect on gonadotropin secretion might be expected. Clomiphene citrate, an oestrogen agonist–antagonist, is clinically employed for induction of ovulation and has been extensively studied for its effect on reproductive function in humans and in rats. In relatively high doses, CC has been shown to reversibly suppress gonadotropin secretion, decrease testosterone levels and cause spermatogenic arrest (Heller *et al.*, 1969; Weissenberg *et al.*, 1992). However, a direct effect of CC on the testis could not be excluded. As shown in our study, this suppression is entirely reversible upon cessation of administration of the drug.

Releasing hormone agonists and gonadal steroid hormones have been evaluated for their capacity to prevent testicular damage caused by cytotoxic drugs. Treatment of mice, rats and baboons with luteinising hormone-releasing hormone (LH-RH) agonists have been reported to confer various degrees of testicular preservation (Glode *et al.*, 1981; Lewis *et al.*, 1985; Ward *et al.*, 1990) notwithstanding contrary views expressed on lack of germinal epithelium protection (da Cunha *et al.*, 1987; Karashima *et al.*, 1988; Papadopoulos, 1991). Several of these studies pointed to suppression of

**Table II** Fertilising ability of male rats following administration of PCB or combined treatment

| | *Group 1* *CC* | | *Group 2* *PCB* | | *Group 3* *CC + PCB* | | *Group 4* *Control* | |
|---|---|---|---|---|---|---|---|---|
| Mating | I | II | I | II | I | II | I | II |
| Fertile (%) | 100 (10/10) | 100 (10/10) | 0 (0/10) | 0 (0/10) | 60 (6/10)* | 80 (8/10)* | 100 (10/10) | 100 (10/10) |
| Size of litter (range) | 8 (7–10) | 11 (10–12) | – | – | 8 (1–11) | 10 (6–12) | 10 (8–12) | 11 (9–12) |

*$P<0.01$ *vs* PCB.

spermatogenesis and prevention of testicular damage in rats undergoing chemotherapy. Moreover, LH-RH analogue failed to protect fertility in men treated with the MOPP regimen for Hodgkin's disease (Johnson *et al.*, 1985; Waxman, 1987). Testosterone was found to protect approximately 22% of seminiferous tubules from damage by PCB in rats (Delic *et al.*, 1986). Oestrogen alone, however, failed to shield the germinal epithelium (Morris and Ward, 1989), but addition of oestradiol to testosterone was found to enhance protection (Parchuri *et al.*, 1993; Meistrich *et al.*, 1994).

Clomiphene might have a direct oestrogenic effect on the testes and therefore acts as androgen antagonist. Direct effects on testicular RNA and protein synthesis have already been demonstrated (Hollinger 1970, 1971; Hollinger and Hwang, 1972). Flickinger (1977) reported alteration of clomiphene on the male reproductive tract similar to those seen following oestrogen treatment. Oestradiol treatment of young rats during 3 weeks caused 'spermatogenic arrest' similar to that observed under clomiphene treatment (R Weissenberg, personal data). Yet, use of oestrogen alone or clomiphene for protection of testis from damage induced by PCB led to contradictory results, for reasons which are not apparent.

### References

CLERMONT Y AND HARVEY SC. (1967). Effects of hormones on spermatogenesis in the rat. In *Endocrinology of the Testis*, Wolstenholme GEW and O'Connor M (eds) pp. 173–189. G&A Churchill: London.

DA CUNHA MF, MEISTRICH ML AND NADER S. (1987). Absence of testicular protection by a gonadotropin-releasing hormone analogue against cyclophosphamid-induced testicular cytotoxicity in the mouse. *Cancer Res.*, **47**, 1093–1097.

DELIC JI, BUSH C AND PECKHAM MJ. (1986). Protection from procarbazine induced damage of spermatogenesis in the rat by androgen. *Cancer Res.*, **46**, 1909–1914.

DRASGA RE, EINHORN LH, WILLIAMS SD, PATEL DN AND STEVENS EE. (1983). Fertility after chemotherapy for testicular cancer. *J. Clin. Oncol.*, **1**, 179-183.

FAIRLEY KF, BARRIE JU AND JOHNSON W. (1972). Sterility and testicular atrophy related to cyclophosphamide therapy. *Lancet*, **i**, 568–569.

FLICKINGER CJ. (1977). Alterations in the male reproductive tract of rats treated with clomiphene. *Am. J. Anat.*, **149**, 533–562.

GLODE LM, ROBINSON J AND GOULD SF. (1981). Protection from cyclophosphamide-induced testicular damage with an analogue of gonadotropin-releasing hormone. *Lancet*, **i**, 1132–1134.

HELLER CG, ROWLEY MJ AND HELLER GV. (1969). Clomiphene citrate; a correlation of its effect on sperm concentration and morphology, total gonadotropins, estrogen and testosterone excretion and testicular cytology in normal men. *J. Clin. Endocrinol.*, **29**, 638–648.

HOLLINGER MA. (1970). Effect of clomiphene on testicular protein synthesis in vitro. *Biochem. Pharmacol.*, **19**, 2701–2705.

HOLLINGER MA. (1971). Study on the inhibitory effect of clomiphene on testicular protein synthesis. *Proc. West Pharmacol. Soc.*, **14**, 101–103.

HOLLINGER MA AND HWANG F. (1972). Effect of in vivo and in vitro administration of clomiphene on RNA synthesis in rat testis. *Arch. Int. Pharmacodyn.*, **197**, 213–221.

JEGOU B, VELEZ DE LA CALLE JF AND BAUNCH F. (1991). Protective effect of medroxyprogesterone acetate plus testosterone against radiation-induced sterility. *Proc. Natl Acad. Sci. USA*, **88**, 8710–8715.

JOHNSON DH, STEIN R, HAINSWORTH JD, LINDE R, VALE W, RIVIER J, FLEXNEN J, WELCH RV, GRECO A. (1985). Effect of luteinizing hormone releasing hormone agonist given during combination chemotherapy on post therapy fertility in male patients with lymphoma. *Blood*, **65**, 832–836.

KARASHIMA T, ZALATNIA A AND SCHALLY AV. (1988). Protective effect of analogues of LhRh against chemotherapy-induced testicular damage in rats. *Proc. Natl Acad. Sci. USA*, **85**, 2329–2333.

KREUSER ED AND HETZEL WD. (1988). Reproductive and endocrine gonadal capacity with and without GnRH analogue application during chemotherapy in patients treated for testicular cancer. In *LhRh Agonists in Oncology*. Hoeffken K (ed.) pp. 111–118. Springer: Berlin.

LEWIS RW, DOWLING KJ AND SCHALLY AV. (1985). D-Tryptophan-6 analog of luteinizing hormone releasing hormone as a protective agent against testicular damage caused by cyclophosphamide in baboons. *Proc. Natl Acad. Sci. USA*, **82**, 2975–2979.

MEISTRICH ML, WILSON G, YE WEI-SAN, KURDOGLU B, PARCHURI N AND TERRY HA. (1994). Hormonal protection from procarbazine-induced testicular damage is selective for survival and recovery of stem spermatogonia. *Cancer Res.*, **54**, 1027–1034.

MORRIS ID AND SHALET SM. (1990). Protection of gonadal function from cytotoxic chemotherapy and irradiation. *Baillière's Clin. Endocrinol. Metab.*, **4**, 97–118.

MORRIS ID AND WARD JA. (1989). Estradiol mediated suppression of testicular function does not alleviate spermatogenic damage resulting from administration of procarbazine. *International Conference on Reproductive and Human Cancer*. Raven Press: New York.

MORRIS ID, BARDIN CW, GUNSALUS G AND WARD GA. (1990). Prolonged suppression of spermatogenesis by oestrogen does not preserve the seminiferous epithelium in procarbazine-treated rats. *Int. J. Androl.*, **12**, 180–189.

NSEYO UO, HUBEN RP, KLIOZE SS AND PONTES JE. (1985). Protection of germinal epithelium with luteinizing hormone analogue. *J. Urol.*, **34**, 187–190.

PAPADOPOULOS I. (1991). LH-RH analogues do not protect the germinal epithelium during chemotherapy. *Urol. Res.*, **19**, 31–34.

PARCHURI N, WILSON G AND MEISTRICH ML. (1993). Protection by gonadal steroid hormones against procarbazine-induced damage to spermatogenic function in LBNF1 hybrid rats. *J. Androl.*, **14**(4), 257–266.

PENNISI AJ, CRUSHKIN CM AND LIEBERMAN E. (1975). Gonadal function in children with nephrosis treated with cyclophosphamide. *Am. J. Dis. Child.*, **129**, 315–318.

ROESER HP, STOCKS AE AND SMITH AJ. (1978). Testicular damage due to cytotoxic drugs and recovery after cessation of therapy. *Aust. NZ. J. Med.*, **8**, 250–254.

SCHILSKY RL, LEWIS BJ, SHERINS RJ AND YOUNG RE. (1980). Gonadal dysfunction in patients receiving chemotherapy for cancer. *Ann. Int. Med.*, **93**, 109–114.

VELEZ DE LA CALLE JF AND JEGOU B. (1990). Protection by steroid contraseptinus against procarbazine-induced sterility and genotoxicity in male rats. *Cancer Res.*, **50**, 1308–1315.

WARD JA, ROBINSON J, FURR BJA, SHALET SM AND MORRIS ID. (1990). Protection of spermatogenesis in rats from the cytotoxic procarbazine by the depot formulation of Zoladex, a gonadotropin-releasing hormone agonist. *Cancer Res.*, **50**, 568–574.

WAXMAN J. (1987). Preserving fertility in Hodgkin's disease. *Baillière's Clin. Hematol.*, **1**, 185–190.

WEISSENBERG R, DAR Y AND LUNENFELD B. (1992). The effect of clomiphene citrate and its Zu or En isomers on the reproductive system of the immature male rat. *Andrologia*, **24**, 161–165.

WHITEHEAD E, SHALET SM, BLACKLEDGE G, TODD I, CROWTHER D AND BEARDWELL C.G. (1982). The effect of Hodgkin's disease and combination chemotherapy on gonadal function in adult male. *Cancer*, **49**, 418–422.